# A comparison of carcass characteristics, carcass cutting yields, and meat quality of barrows and gilts

**DOI:** 10.1093/tas/txad079

**Published:** 2023-07-13

**Authors:** Benjamin M Bohrer, Justice B Dorleku, Cheryl P Campbell, Marcio S Duarte, Ira B Mandell

**Affiliations:** Department of Animal Sciences, The Ohio State University, Columbus, Ohio 43210, USA; Department of Food Science, University of Guelph, Guelph, ON, CanadaN1G 2W1; Department of Animal Biosciences, University of Guelph, Guelph, ON, CanadaN1G 2W1; Department of Animal Biosciences, University of Guelph, Guelph, ON, CanadaN1G 2W1; Department of Animal Biosciences, University of Guelph, Guelph, ON, CanadaN1G 2W1

**Keywords:** lean yield, pork carcass, pork quality, primal pass rate

## Abstract

Objectives of this research were to compare carcass characteristics, carcass cutting yields, and meat quality for market barrows and market gilts. Commercially-sourced carcasses from 168 market barrows and 175 market gilts weighing an average of 107.44 ± 7.37 kg were selected from 17 different slaughter groups representing approximately 3,950 carcasses. Each group was sorted into percentiles based on hot carcass weight with an equal number of barrows and gilts selected from each quartile so that weight minimally confounded parameters of interest. Carcass lean yield was determined for carcasses following fabrication (i.e. dissection of lean, fat, and bone tissue components) and meat quality measurements were evaluated at the time of fabrication (24 to 72 h postmortem) and following 14-d of postmortem storage. Data were analyzed as a randomized complete block design with carcass serving as the experimental unit, sex (barrow or gilt), the three hot carcass weight quantiles (light [<104 kg]; average [104 to 110 kg]; heavy [>110 kg]), and the interaction between sex and hot carcass weight quantile serving as fixed effects, and producer nested within slaughter event serving as a random effect. Results from the study demonstrated that gilt carcasses were leaner (3 mm less backfat thickness; 3.5 cm^2^ greater loin muscle area, 1.52% greater merchandized-cut yield, and 2.92% greater dissected carcass lean yield; *P* < 0.01) than barrow carcasses, while loins from barrows were higher quality (0.43% more intramuscular fat and slightly less shear force; *P* < 0.01) than loins from gilts. While this study confirms the well-known biological principle that barrow carcasses have greater levels of fat deposition and lower levels of carcass leanness when compared with gilt carcasses, this study provides a much-needed quantification of these differences for the commercial industry that will undoubtedly be useful as new technologies emerge in upcoming years.

## Introduction

Previous research has successfully characterized most attributes associated with the differences in live performance, carcass composition, and meat quality between conventionally-castrated barrows (i.e. male market pigs undergoing surgical castration prior to weaning; described as *barrows* throughout this manuscript) and conventionally-raised gilts (i.e. female market pigs managed without suppression of estrus; described as *gilts* throughout this manuscript) (Bereskin and Davey, 1978; Martel et al., 1988; Uttaro et al., 1993; Unruh et al., 1996; Lee et al., 2013; Boler et al., 2014; Kyle et al., 2014; Smit et al., 2014; Davis et al., 2015; Overholt et al., 2016; Lowell et al., 2019; Redifer et al., 2020). Most recently, a 34-study meta-analysis reported barrows were associated with 5.9% greater average daily gain, 11.4% greater average daily feed intake, 4.3% lower (i.e. poorer) feed efficiency (gain to feed), 11.7% greater backfat thickness, 4.5% lower predicted lean yield, and 15.2% greater marbling when compared with gilts (Woodworth et al., 2021). This is significant information for the industry since approximately half of pork production systems in most countries of the world are comprised of barrows, while the other half is comprised of gilts. Narrowing production and processing gaps between barrows and gilts should be prioritized by the pork industry globally, as differences between barrows and gilts is a significant driver of the variation present in various sectors of pork production systems (Rodríguez-Sánchez et al., 2011; Overholt et al., 2016; Arkfeld et al., 2017).

It was hypothesized that gilts would be leaner than barrows, which has been well documented by previous research (Boler et al., 2014; Overholt et al., 2016; Redifer et al., 2020). However, the effect of carcass weight is often considered a confounding factor for carcass leanness in research studies and is a factor that can be improved in the commercial setting with the use of multiple marketing groups (Arkfeld et al., 2016; Zhou and Bohrer, 2019). Based on the assumption that gilts have a 5.9% reduction in average daily gain compared with barrows, differences attributed to the main effect of sex for final live weight and hot carcass weight could be 3.0 and 2.3 kg, respectively, when pigs are marketed at industry-standard market weights on a fixed-time basis (Woodworth et al., 2021). Yet, as mentioned, most commercial producers work to narrow these production differences by using multiple marketing groups, thus allowing slower-growing pigs greater time on feed to reach targeted end weights. Nonetheless, it has been well documented that lighter-weight carcasses are trimmer than heavier-weight carcasses (Ohlmann and Jones, 2011; Plà-Aragonés et al., 2013; Rodríguez et al., 2014; Price et al., 2019; Barducci et al., 2020), so reducing the confounding effect of carcass weight could prove to be useful when evaluating carcass cutting yields and meat quality between barrows and gilts. The objectives of this research were to compare carcass characteristics, carcass cutting yields, and meat quality for market barrows and market gilts.

## Materials and Methods

Commercial pigs were slaughtered under the supervision of the Canadian Food Inspection Agency (**CFIA**) at a federally inspected processing facility. Meat samples were obtained from the processing facility; therefore, no Institutional Animal Care and Use Committee approval was necessary for this study.

### Packing Plant Data Collection

Pigs were sourced from nine different producers and slaughtered at the same commercial pork processing facility over a 9-month period. While a wide range of commercial genetic lines was represented in this study, most pigs used for this study were crossbred progeny from Duroc sires and commercial white line (Large White × Landrace) dams. A total of 17 slaughter events consisting of approximately 3,950 carcasses were utilized to select the 343 carcasses that were used in the study.

Pigs had at least 8 h of lairage with access to water but no access to feed. Slaughter was conducted using a low-stress driving system with green lights to elicit a shadow-free environment and pigs were moved in groups of 6 to 8 pigs with a hydraulic gate push system into a CO_2_ stunning system (i.e. Frontmatec CO_2_ stunning system, Kolding, Frontmatec). Stunning was immediately followed by exsanguination, scalding, and dehairing in accordance with the standard operating procedures for the commercial pork packing plant. Individual carcass identification began after the pigs were dehaired and was maintained throughout the slaughter and chilling process. Carcass identification was completed prior to carcasses being scanned by an automated ultrasonic scanner (AutoFom III, Smørum, Frontmatec). Sex (barrow or gilt), carcass ID, and sequence number for each carcass were recorded as carcasses were being individually scanned with the automated ultrasonic carcass scanner. Carcasses were then subjected to a singeing tunnel, a brush tunnel, evisceration, and splitting (heads remained with carcasses). The ventral side of carcasses was split using a robotic breast and belly opener (AiRA RBO Breast and Belly Opener; Kolding, Frontmatec) while the dorsal side of carcasses was split with a robotic splitter saw (AiRA RPS-S Splitter with Saw; Kolding, Frontmatec). Accuracy of carcass splits was determined using the following scale to determine split uniformity: 1 = ideal (split from tail to neck along the mid-line of the spinal column into complete symmetrical halves); 2 = acceptable (split from tail to neck along the mid-line of the spinal column into moderately symmetrical halves); 3 = marginally acceptable (split from tail to neck along the mid-line of the spinal column into slightly equal halves); *x* = unacceptable (split from tail to neck along the mid-line of the spinal column into unequal halves).

After dressing of the carcasses was completed (i.e. carcasses passed the final CFIA inspection at the last rail-out location), study personnel recorded the order of the carcasses on the moving line along with examining each carcass for evidence of carcass defects or missing components. Examples of carcass defects (i.e. missing components) that were noted throughout the study included missing feet, missing legs, missing head, over scalding of the carcass resulting in trim, loss of ear(s), and removal of skin, fat, or lean (that normally would be kept on the carcass during carcass dressing).

Grading probe measurements (backfat thickness and loin muscle depth) were collected online by experienced operators from the Ontario Pork Grading Authority using a Destron PG-100 probe (International Destron Technologies). The handheld grading probe was inserted perpendicularly at the grading site between the third and fourth last ribs, 7 cm off the split-line according to Canadian grading standards (Pomar and Marcoux, 2003). Backfat thickness and muscle depth (i.e. loin depth) measurements were used to obtain the predicted lean yield value for each carcass using the following two equations (CPC, 1994; Bohrer et al., 2023):


$$\text{Destron predicted lean yield }(1994\text{ equation})=68.1863-(0.7833\times \text{backfat thickness})+(0.0689\times \text{muscle depth})+(0.0080\times {\text{backfat thickness}}^{2})-(0.0002\times {\text{muscle depth}}^{2})+(0.0006\times \text{backfat thickness}\times \text{muscle depth})$$

$$\begin{array}{l} \text{Destron predicted lean yield (2023 equation)=89.16298}-\text{(1.63023}\times \text{backfat thickness)}\\ -\text{(0.42126}\times \text{muscle depth)}\\ +\text{(0.01930}\times {\text{backfat thickness}}^{\text{2}}\text{)}\\ \text{+(0.00308}\times {\text{muscle depth}}^{\text{2}}\text{)}\\ \text{+(0.00369}\times \text{backfat thickness}\times \text{muscle depth)} \end{array}$$

where backfat thickness (mm) and the muscle depth (mm) are collected at the grading site for each carcass.

Hot carcass weight was recorded at the grading station location. Following the grading station, carcasses were chilled using a conventional chilling system, which can be described as a reefer cooler (using ammonia) operating on a specific chill cycle that is proprietary to the commercial packing plant with carcasses chilled for 22 to 24 h at ambient temperatures ranging from −4 to 2 °C.

### Selection of Carcasses for Cutting Yield Tests

For each of the 17 slaughter events, approximately 20 carcasses were selected to be used in this study. Selection priorities focused on being representative of the pigs slaughtered in the commercial setting while equally representing barrows and gilts across the different weights.

For each slaughter event, data for individual carcasses (i.e. sex, grading probe measurements, split scores, hot carcass weight, and presence of carcass defects [i.e. missing components]) were initially entered into Microsoft Excel (Microsoft Corporation; Redmond, Washington, USA) and then sorted by sex into two groups (barrows or gilts) and by hot carcass weight into percentiles based off of population averages. Once sorting was completed, three or four barrow carcasses and three or four gilt carcasses were selected from each carcass weight quartile in an equivalent manner (i.e. equal numbers of barrow and gilt carcasses were selected from each weight quartile). Finally, backfat thickness was arranged into three groups (≤15 mm, 15.5 to 19 mm, ≥19.5 mm), and efforts were made to equally represent trim, intermediate, and fat carcasses among each of the sex groups. Carcasses with defects (i.e. missing components) or unacceptable splits (i.e. carcass split score 3 or carcass split score *x*) were not considered for use in the study.

The left sides of selected carcasses were placed in meat combos (i.e. plastic bag lined 8-sided cardboard bulk meat containers placed on top of a wooden pallet) approximately 20 to 24 h following slaughter. Combos were immediately delivered to the University of Guelph Meat Laboratory using a refrigerated truck that met CFIA guidelines for transportation.

### Carcass Fabrication

Upon arrival to the university meat laboratory, (approximately 24 to 28 h postmortem), pork carcass sides were removed from combos, hung on an overhead rail, and refrigerated at ≤4 °C until fabrication. Carcass sides were individually weighed and fabricated into primals (ham, picnic shoulder, butt shoulder, belly, and loin) and subprimals according to Institutional Meat Purchase Specifications (NAMP, 2006; IMPS, 2014) guidelines at 24 to 72 h postmortem. Immediately after weights of primals and subprimals were collected, subprimals were then further separated into lean, fat, and bone components.

### Lean Yield Equations

Dissected lean yield was calculated for each of the primals (ham, picnic shoulder, butt shoulder, belly, and loin) by dividing the lean components of the primals by the untrimmed primal weights. Three cutting yield equations were used to capture untrimmed primal cutting yields, merchandized cut cutting yields, and dissected carcass lean yield:


Primal−cut yield=[(IMPS #401A ham+IMPS #405 picnic+IMPS #406 butt+IMPS #408B belly +IMPS #410 loin)/chilled side weight]×100.

$$\begin{array}{l} \text{Merchandized-cut yield}=\text{\{[(IMPS \#402G ham}+\text{additional lean from ham)+}\\ \text{IMPS \#405A picnic + IMPS \#406A butt + IMPS \#408 belly+}\\ \text{IMPS \#416 spareribs + IMPS \#413D sirloin + IMPS \#414 Canadian back loin+}\\ \text{IMPS \#415 tenderloin + IMPS \#422 back ribs]/chilled side weight\} }\times \text{100}\text{.} \end{array}$$

**Dissected carcass lean yield** = [(dissected lean from ham + dissected lean from picnic + dissected lean from butt + dissected lean from belly + dissected lean from loin + dissected lean from ribs and neckbones) / chilled side weight] × 100.

### Loin Measurements

During fabrication, bone-in loins were cut at the Canadian grading site location (between the third and fourth last rib). This location was identified with the aid of the punctures on the carcass made by the Destron probe during carcass grading. Backfat thickness and muscle depth were measured with a ruler and loin eye area was measured by tracing the perimeter of the *longissimus* muscle on acetate paper and then quantified using an electronic planimeter (Manually Operated Planimeter III; Carl Zeiss Inc., Thornwood, New York, USA).

Anterior to the Canadian grading site (toward the blade end of the loin), 3.0 cm-thick boneless loin chops were cut, trimmed of external fat, and the *longissimus thoracis* muscle was separated from other muscles. A series of analyses were performed on the *longissimus thoracis* muscle samples including the assessment of pH, drip loss via EZ cup methodology, instrumental color, intramuscular fat content via lipid extraction, subjective color, subjective marbling, subjective firmness, Warner-Bratzler shear force, and cooking loss.

pH was measured 24 to 72 h postmortem (at the time of fabrication) at three different locations on the same loin chop using a spear-tipped pH meter (Hanna HI98163; Hanna Instruments, Mississauga, Ontario, Canada). The pH meter was calibrated with buffer solutions that were stored at refrigerated temperatures (≤4 °C) prior to use and then checked using the buffer solutions at incremental time points during use.

Drip loss was measured over a 48-h period in samples that were 24to 72 h postmortem (at the time of fabrication) at the beginning of the measurement using two 25-mm diameter cores with the EZ cup method described previously by Rassmussen and Andersson (1996).

Instrumental color was evaluated 24 to 72 h postmortem (at the time of fabrication) and following 14 d of postmortem storage (samples were wet-aged in a vacuum-sealed package and stored at refrigerated temperatures in the dark) with a calibrated, handheld Minolta CR-400 Chroma meter (Konica Minolta Sensing Americas, Inc., Ramsey, New Jersey, USA) with illuminant D_65_, an 8-mm aperture, and 0° viewing angle settings. Samples were allowed to bloom for a period of 30 min prior to evaluation and each measurement by the Chroma meter was reported using the L*, a*, and b* color space. Two measurements per sample were collected and then averaged to determine instrumental color values for each sample at each time point.

Intramuscular fat (**IMF**) content was determined using the Ankom XT20 Fat Analyzer for ether extraction of fat (AOAC, 2016). A homogenized loin chop was freeze-dried, and then a ground 2.0-g sample was extracted with petroleum ether for 30 min (AOAC, 2016).

Subjective color, marbling, and firmness were evaluated 24 to 72 h postmortem (at the time of fabrication) using NPPC subjective scoring standards (color scores: 1 = pale pinkish gray to white, 2 = grayish pink, 3 = reddish pink, 4 = dark reddish pink, 5 = purplish red, 6 = dark purplish red; marbling scores: 1 through 10 corresponding with intramuscular lipid content; firmness scores: 1 = soft, cut surface distorts easily, 2 = firm, cut surface tends to hold shape, 3 = very firm, cut surface were very smooth with no distortion of shape; National Pork Producers Council, 2000). The same trained individual assessed subjective color, marbling, and firmness throughout the duration of the study, and this individual was blind to treatment at the time of the evaluations.

Warner-Bratzler shear force and cooking loss were determined for samples following both 24 to 72 h of postmortem aging (at the time of fabrication) and 14 d of postmortem storage (samples were wet-aged in a vacuum-sealed package and stored at refrigerated temperatures in the dark) using methodology previously described by Streiter et al. (2012). Briefly, chops were thawed and weighed before being cooking to an internal temperature of 72 °C on a clamshell Garland Grill (Ed-30B: Garland Commercial Ranges Ltd, Mississauga, ON) set to a surface temperature of 105 °C. Following cooking, samples were cooled in a refrigerator to an internal temperature of approximately 4 °C. Six to eight 1.25-cm diameter cores running parallel to the muscle fibers were removed from each sample. Each core was sheared perpendicularly to the muscle fibers with a Warner-Bratzler blade using a TA-XT Plus Texture Analyzer (Texture Technologies Corp., Scarsdale, New York, USA) at a crosshead speed of 3.3 mm/s. The average peak force was recorded for each sample.

### Statistical Analyses

As mentioned above, carcasses were selected based on sex (barrow or gilt) and hot carcass weight quartiles (i.e. equal numbers of barrow and gilt carcasses were selected from each weight quartile). Once data were collected, it was determined that using three quantiles rather than four quartiles generated a more logical and applicable dataset. Thus, data (*N* = 337) were analyzed as a randomized complete block design with sex (barrow or gilt), the three hot carcass weight quantiles (light [<104 kg]; average [104 to 110 kg]; heavy [>110 kg]), and the interaction between sex and hot carcass weight quantile as fixed effects and producer nested within slaughter event as a random effect. Carcass was designated as the experimental unit for all analyses. All data were analyzed using the GLIMMIX procedure in SAS version 9.4 (SAS Institute Inc. Cary, North Carolina, USA). Least squares means were separated using the PDIFF option in SAS and a Tukey–Kramer adjustment was utilized to protect against committing Type-1 statistical errors. To address the objectives of this study, results for the main effect of sex were presented and discussed, whereas the interactions between sex and hot carcass weight quantile were presented as supplementary tables. For discussion purposes, results were considered significant at *P* ≤ 0.05 and the standard error of the difference (**SED**) values were provided.

## Results and Discussion

### Carcass Characteristics

Hot carcass weight and chilled side weight were not different (*P* ≥ 0.24) between barrows and gilts in this study (Table 1). This was expected based on the selection criteria used in this study and assisted in addressing the objectives of this study. However, it is worth mentioning that barrows are expected to be heavier than gilts in fixed-time grow-finish production scenarios. Specifically, Woodworth et al. (2021) reported barrows grow 5.9% faster than gilts which was associated with a difference in carcass weights of approximately 2.3 kg.

**Table 1.** Effect of sex (conventionally-raised market barrows versus conventionally-raised market gilts) on carcass characteristics

	Barrows	Gilts	Effect (barrows − gilts)	95% CI	SED	*P*-value
No. of observations	168	175				
Hot carcass weight^1^, kg	107.49	107.07	0.42	(−0.27, 1.11)	0.35	0.24
Chilled side weight, kg	47.35	47.31	0.04	(−0.29, 0.36)	0.16	0.84
Backfat thickness (Destron probe^2^), mm	18.52	15.67	2.85	(2.05, 3.65)	0.41	<0.01
Muscle depth (Destron probe^2^), mm	67.95	69.60	−1.65	(−3.01, −0.30)	0.69	0.02
Predicted lean yield (Destron 1994 equation^2^), %	61.09	62.45	−1.36	(−1.74, −0.98)	0.19	<0.01
Predicted lean yield (Destron 2023 equation^2^), %	56.34	58.33	−1.99	(−2.56, −1.44)	0.28	<0.01
Backfat thickness (ultrasound^3^), mm	17.96	15.21	2.75	(2.05, 3.45)	0.35	<0.01
Muscle depth (ultrasound^3^), mm	66.67	70.14	−3.47	(−4.75, −2.22)	0.64	<0.01
Backfat thickness (ruler^4^), mm	18.62	15.47	3.15	(2.28, 4.00)	0.44	<0.01
Muscle depth (ruler^4^), mm	69.28	71.93	−2.65	(−3.69, −1.59)	0.53	<0.01
Loin eye area (tracing^4^), cm^2^	54.89	58.34	−3.45	(−4.56, −2.33)	0.56	<0.01

^1^Hot carcass weight was measured as a head-on weight.

^2^The optical probe used in this study was the Destron PG-100 (International Destron Technologies); predicted lean yield equations were based on the following references (CPC, 1994; Bohrer et al., 2023).

^3^The ultrasound technology used in this study was the AutoFom III (Frontmatec A/S).

^4^Ruler and tracing measurements were collected on chilled carcasses during fabrication.

Carcass leanness parameters (backfat thickness, muscle depth, loin eye area, and predicted lean yield) were significantly different (*P* < 0.05) among barrows and gilts in this study regardless of the technique used to collect measurements. When using the Destron optical grading probe, backfat thickness was 2.85 mm greater, muscle depth was 1.65 mm less, and predicted lean yield was 1.36% less when using the 1994 equation and 1.99% less when using the 2023 equation for barrows compared with gilts. When using the AutoFom III ultrasonic carcass scanner backfat thickness was 2.75 mm greater and muscle depth was 3.47 mm less for barrows compared with gilts. When collecting measurements with the ruler (in the case of backfat thickness a muscle depth) or the planimeter (in the case of loin eye area), backfat thickness was 3.15 mm greater, muscle depth was 2.65 mm less, and loin eye area was 3.45 cm^2^ less for barrows compared with gilts. These are not new findings, as several other recent research studies (Boler et al., 2014; Overholt et al., 2016; Redifer et al., 2020) have reported there are leanness advantages for gilt carcasses when compared with barrow carcasses. However, there has not been a recent study conducted at current carcass weights and with modern genetics that has evaluated carcass cutting yields and dissected lean yields between barrow and gilt carcasses. In addition, most previous analyses have been confounded by carcass weight differences between barrows and gilts, which was reduced when considering the selection criteria used in this study.

### Primal Cut-Out Yields

#### Ham primal

The 401A whole ham (untrimmed, bone-in ham) was 0.26 kg lighter (*P* < 0.01; SED = 0.07 kg) and made up 0.56% less of the side weight (*P* < 0.01; SED = 0.12%) for barrows compared with gilts (Table 2). The 401C trimmed ham (skinless, trimmed, semi-boneless ham) was 0.45 kg lighter (*P* < 0.01; SED = 0.08 kg) and made up 0.98% less of the side weight (*P* < 0.01; SED = 0.15%) for barrows compared with gilts. The 402G lean ham + additional lean from the ham primal (3-muscle boneless ham with additional lean) was 0.49 kg lighter (*P* < 0.01; SED = 0.07 kg) and made up 0.56% less of the side weight (*P* < 0.01; SED = 0.12%) for barrows compared with gilts. Dissected lean yield of the ham was 2.52% lower (*P* < 0.01; SED = 0.29%) for barrows compared with gilts. Overall, this study concluded that hams from barrows were lighter and lower yielding than hams from gilts.

**Table 2.** Effect of sex (conventionally-raised market barrows versus conventionally-raised market gilts) on whole and trimmed ham primal cut-out values

	Barrows	Gilts	Effect (barrows − gilts)	95% CI	SED	*P*-value
Whole ham (401A), kg	12.85	13.11	−0.26	(−0.40, −0.12)	0.07	<0.01
% chilled side weight	27.16	27.72	−0.56	(−0.81, −0.32)	0.12	<0.01
Trimmed ham (401C), kg	9.63	10.08	−0.45	(−0.61, −0.31)	0.08	<0.01
% chilled side weight	20.35	21.33	−0.98	(−1.26, −0.69)	0.15	<0.01
Lean ham (402G + additional lean), kg	8.17	8.66	−0.49	(−0.63, −0.35)	0.07	<0.01
% chilled side weight	27.16	27.72	−0.56	(−0.81, −0.32)	0.12	<0.01
Ham dissected lean yield^1^, %	63.49	66.01	−2.52	(−3.08, −1.94)	0.29	<0.01

^1^Ham dissected lean yield = [(lean from 402G ham + additional lean)/401A ham] × 100.

#### Shoulder primals

The 403 whole shoulder + neckbones (untrimmed, foot-removed, bone-in shoulder) was 0.11 kg heavier (*P* = 0.03; SED = 0.05 kg) and made up 0.22% more of the side weight (*P* = 0.01; SED = 0.08%) for barrows compared with gilts (Table 3). The 405 picnic (skin-on, semi-boneless picnic) and the 405A boneless picnic (skinless, boneless picnic) were not different (*P* ≥ 0.12) in their weights or their percentages of side weight when comparing barrows and gilts. The 406 butt (skin-on, semi-boneless butt) was 0.09 kg heavier (*P* < 0.01; SED = 0.04 kg) and made up 0.17% more of the side weight (*P* < 0.01; SED = 0.05%) for barrows compared with gilts. The 406A boneless butt (skinless, boneless butt) was not different (*P* ≥ 0.09) in weight or percentage of side weight when comparing barrows and gilts. Despite the low magnitude of difference for the boneless picnic and boneless butt weights, large differences existed for fat-free lean in the shoulder primals. Dissected lean yield of the picnic was 2.73% lower (*P* < 0.01; SED = 0.31%) for barrows compared with gilts. Dissected lean yield of the butt was 3.77% lower (*P* < 0.01; SED = 0.45%) for barrows compared with gilts. Dissected lean yield of the whole shoulder was 2.94% lower (*P* < 0.01; SED = 0.32%) for barrows compared with gilts. Overall, this study concluded that untrimmed bone-in shoulder cuts from barrows were slightly heavier and slightly higher yielding than untrimmed bone-in shoulder cuts from gilts; however, it was revealed that shoulder cuts from barrows were much lower yielding once trimmed and evaluated for leanness when compared with shoulder cuts from gilts.

**Table 3.** Effect of sex (conventionally-raised market barrows versus conventionally-raised market gilts) on whole and trimmed shoulder primal cut-out values

	Barrows	Gilts	Effect (barrows − gilts)	95% CI	SED	*P*-value
Whole shoulder (403 + neckbones), kg	9.43	9.32	0.11	(0.01, 0.21)	0.05	0.03
% chilled side weight	19.91	19.69	0.22	(0.06, 0.39)	0.08	0.01
Picnic (405), kg	4.79	4.76	0.03	(−0.03, 0.09)	0.03	0.32
% chilled side weight	10.11	10.06	0.05	(−0.05, 0.16)	0.05	0.28
Boneless picnic (405A), kg	4.32	4.35	−0.03	(−0.09, 0.02)	0.03	0.19
% chilled side weight	9.12	9.20	−0.08	(−0.19, 0.02)	0.05	0.12
Butt (406), kg	4.65	4.56	0.09	(0.03, 0.14)	0.04	<0.01
% chilled side weight	9.81	9.64	0.17	(0.07, 0.28)	0.05	<0.01
Boneless butt (406A), kg	3.67	3.71	−0.04	(−0.09, 0.01)	0.03	0.16
% chilled side weight	7.75	7.83	−0.08	(−0.18, 0.01)	0.05	0.09
Picnic dissected lean yield^1^, %	66.87	69.60	−2.73	(−3.33, −2.13)	0.31	<0.01
Butt dissected lean yield^2^, %	63.08	66.85	−3.77	(−4.65, −2.90)	0.45	<0.01
Shoulder dissected lean yield^3^, %	61.48	64.42	−2.94	(−3.58, −2.30)	0.32	<0.01

^1^Picnic dissected lean yield = (lean from 405A boneless picnic/405 picnic) × 100.

^2^Butt dissected lean yield = (lean from 406A boneless butt/406 butt) × 100.

^3^Shoulder dissected lean yield = [(lean from 405A boneless picnic + lean from 406A boneless butt + lean from neckbones)/(403 shoulder + neckbones)] × 100.

#### Belly primal

The 408B whole belly (skin-on, untrimmed, bone-in belly), the 408 boneless belly (skinless, untrimmed, boneless belly), and the 416 spareribs (untrimmed spareribs) were not different (*P* ≥ 0.08) in their weights or their percentages of side weight when comparing barrows and gilts (Table 4). However, dissected lean yield of the belly was 2.26% lower (*P* < 0.01; SED = 0.51%) for barrows compared with gilts. Overall, this study concluded that the belly primal and merchandized cuts from the belly primal were not different between barrows and gilts. However, there were significant differences in dissected lean yield of the belly with barrows having lower values than gilts. This is likely an advantage for barrows when compared with gilts as belly processors and consumers typically prefer to work with fatter bellies as opposed to leaner bellies (Person et al., 2005; Soladoye et al., 2015).

**Table 4.** Effect of sex (conventionally-raised market barrows versus conventionally-raised market gilts) on whole and trimmed belly primal cut-out values

	Barrows	Gilts	Effect (barrows − gilts)	95% CI	SED	*P*-value
Whole belly (408B), kg	8.71	8.65	0.06	(−0.06, 0.19)	0.06	0.31
% chilled side weight	18.37	18.27	0.10	(−0.11, 0.33)	0.11	0.34
Boneless belly (408), kg	6.44	6.34	0.10	(−0.01, 0.21)	0.06	0.08
% chilled side weight	13.57	13.38	0.19	(−0.02, 0.40)	0.11	0.08
Spareribs (416), kg	1.44	1.42	0.02	(−0.01, 0.04)	0.01	0.31
% chilled side weight	3.04	3.01	0.03	(−0.03, 0.08)	0.03	0.35
Belly dissected lean yield^1^, %	51.17	53.43	−2.26	(−3.27, −1.26)	0.51	<0.01

^1^Belly dissected lean yield = [(lean from 409A belly + lean from 416 spareribs)/ 408B belly] × 100.

#### Loin primal

The 410 skin-on whole loin (skin-on, untrimmed, bone-in loin), 413D boneless sirloin (skinless, trimmed, boneless sirloin), 415 tenderloin (trimmed tenderloin), and 422 back ribs (untrimmed back ribs) were not different (*P* ≥ 0.07) in their weights or their percentages of side weight when comparing barrows and gilts (Table 5). The 410 skinless whole loin (skinless, untrimmed, bone-in loin) was 0.32 kg lighter (*P* < 0.01; SED = 0.06 kg) and made up 0.69% less of the side weight (*P* < 0.01; SED = 0.11%) for barrows compared with gilts. The 414 Canadian back loin (trimmed boneless loin) was 0.24 kg lighter (*P* < 0.01; SED = 0.04 kg) and made up 0.51% less of the side weight (*P* < 0.01; SED = 0.07%) for barrows compared with gilts. Dissected lean yield of the loin was 3.90% lower (*P* < 0.01; SED = 0.45%) for barrows compared with gilts. Overall, this study concluded that the untrimmed loin primal and several merchandized cuts from the loin primal including the sirloin, tenderloin, and back ribs were not different between barrows and gilts. However, there were significant differences between barrows and gilts once loins were trimmed as trimmed loins and boneless loins from barrows were lighter and lower yielding than those pieces from gilts. In addition, there was a large difference in dissected lean yield of the loin between barrows and gilts, with barrows having nearly a 4% lower yield compared with gilts. This large magnitude of difference between barrows and gilts would generally be viewed by the industry as a negative for barrows; however, as discussed in a later section, the fatter loins associated with barrows may be responsible for some of the observed advantages in meat quality traits of barrows versus gilts such as intramuscular fat content. In addition, if bone-in loins are being merchandized, excessively trim loins could create difficulties for processors during the skinning process and be less desirable for processors even when considering the lower lean yield associated with excessively fat loins.

**Table 5.** Effect of sex (conventionally-raised market barrows versus conventionally-raised market gilts) on whole and trimmed loin primal cut-out values.

	Barrows	Gilts	Effect (barrows − gilts)	95% CI	SED	*P*-value
Whole loin, skin-on (410), kg	13.79	13.67	0.12	(−0.03, 0.28)	0.08	0.13
% chilled side weight	29.13	28.88	0.25	(−0.02, 0.51)	0.13	0.07
Whole loin, skinless (410), kg	10.70	11.02	−0.32	(−0.45, −0.19)	0.06	<0.01
% chilled side weight	22.60	23.29	−0.69	(−0.91, −0.47)	0.11	<0.01
Boneless sirloin (413D), kg	1.50	1.46	0.04	(−0.01, 0.08)	0.02	0.12
% chilled side weight	3.16	3.09	0.07	(−0.02, 0.16)	0.04	0.13
Canadian back loin (414), kg	3.61	3.85	−0.24	(−0.31, −0.17)	0.04	<0.01
% chilled side weight	7.62	8.13	−0.51	(−0.65, −0.36)	0.07	<0.01
Tenderloin (415), kg	0.88	0.89	−0.01	(−0.03, 0.01)	0.01	0.59
% chilled side weight	1.86	1.88	−0.02	(−0.05, 0.03)	0.02	0.50
Back ribs (422), kg	2.32	2.35	−0.03	(−0.07, 0.01)	0.02	0.11
% chilled side weight	4.92	4.98	−0.06	(−0.14, 0.01)	0.03	0.11
Loin dissected lean yield^1^, %	53.21	57.11	−3.90	(−4.79, −3.02)	0.45	<0.01

^1^Loin dissected lean yield = [(lean from 413D sirloin + lean from 414 Canadian back loin + lean from 415 tenderloin + lean from 422 back ribs)/ 410 loin skin-on] × 100.

#### Cut yield equations

Primal-cut yield in this study, which is the summation of untrimmed primal cuts (401A whole ham, 405 picnic, 406 butt, 408B whole belly, and 410 skin-on whole loin) divided by chilled side weight, was not different (*P* = 0.58) between barrows and gilts (Table 6). Boler et al. (2014) previously reported a difference for carcass cutting yield (summation of trimmed ham, trimmed shoulder, trimmed belly, and trimmed loin divided by chilled side weight) between barrows and gilts, with barrows having 0.92% lower carcass cutting yield compared with gilts. This could be attributed to the differences that begin to occur between barrow and gilt carcasses with trimming.

**Table 6.** Effect of sex (conventionally-raised market barrows versus conventionally-raised market gilts) on carcass cutting yields

	Barrows	Gilts	Effect (barrows − gilts)	95% CI	SED	*P*-value
Primal-cut yield^1^, %	94.57	94.55	0.02	(−0.06, 0.10)	0.04	0.58
Merchandized-cut yield^2^, %	68.30	69.82	−1.52	(−1.86, −1.17)	0.18	<0.01
Dissected carcass lean yield^3^, %	55.44	58.36	−2.92	(−3.61, −2.22)	0.35	<0.01

^1^Primal-cut yield = [(401A ham + 405 picnic + 406 butt + 408B belly + 410 loin)/chilled side weight] × 100.

^2^Merchandized-cut carcass cutting yield = {[(402G ham + additional lean) + 405A picnic + 406A butt + 408 belly + 416 spareribs + 413D sirloin + 414 Canadian back loin + 415 tenderloin + 422 back ribs]/chilled side weight} × 100.

^3^Dissected carcass lean yield = [(dissected lean from ham + dissected lean from picnic + dissected lean from butt + dissected lean from belly + dissected lean from loin + dissected lean from ribs and neckbones)/chilled side weight] × 100.

Merchandized-cut yield in this study, which is the summation of the fabricated cuts that are typically prepared by the packing industry for further processing or sold directly to the end-user divided by chilled side weight, was 1.52% less (*P* < 0.01; SED = 0.18%) for barrows compared with gilts. Boler et al. (2014) previously reported a significant difference for boneless carcass cutting yield (summation of fabricated/merchandized cuts divided by chilled side weight) between barrows and gilts, with barrows having 1.00% lower carcass cutting yields compared with gilts. This further underlines the differences between carcasses from barrows and gilts during trimming/fabrication.

Dissected carcass lean yield in this study, a measure of separated lean throughout the entire process of meat dissection, was 2.92% less (*P* < 0.01; SED = 0.35%) for barrows compared with gilts. The Boler et al. (2014) study did not evaluate dissected carcass lean yield or carcass fat-free lean yield and there has been limited evaluation of these parameters in recent studies. There are several studies, all of which are over 20 yr old, that have evaluated dissected carcass lean yield and/or carcass fat-free lean yield between barrows and gilts. Those studies require interpretation with caution as genetics and carcass weights have changed drastically over the past 20 yr. Notably, Hicks et al. (1998) evaluated carcass fat-free lean yield from seven different genotypes of market barrows and gilts slaughtered at an average carcass weight of 83 kg. That study (Hicks et al., 1998) reported the average difference for carcass fat-free lean yield between barrows and gilts as 3.66% (with a range 0.58% to 5.87% depending on the genotype). This highlights the large variation in the magnitude of change for dissected carcass lean yield and/or carcass fat-free lean yield between different populations of barrows and gilts.

### Loin Quality and Pass-Rate

Loin pH, L* (lightness), a* (redness), subjective color, subjective firmness, and cooking loss were not different (*P* ≥ 0.06) between barrows and gilts in this study (Table 7). Drip loss was slightly lower (*P* = 0.02; SED = 0.44%) for barrows compared with gilts. b* (yellowness) at both aging points evaluated in this study was slightly greater (*P* < 0.05) for barrows compared with gilts. IMF percentage was 0.43% greater (*P* < 0.01; SED = 0.08%) and subjective marbling was 0.27 units greater (*P* < 0.01; SED = 0.08 units) for barrows compared with gilts. This difference aligns with the meta-analysis by Woodworth et al. (2021), which reported that loins from barrows had 15.2% greater marbling (or roughly greater 0.45 marbling score units when assuming average marbling scores for loins is 3.0) than loins from gilts. Warner-Bratzler shear force at both aging times evaluated in this study was slightly less (*P* ≤ 0.01) for barrows compared with gilts. This contrasts with a recent study by Redifer et al. (2020), which reported Warner-Bratzler shear force was not different between barrows and gilts.

**Table 7.** Effect of sex (conventionally-raised market barrows versus conventionally-raised market gilts) on loin quality traits

	Barrows	Gilts	Effect (barrows − gilts)	95% CI	SED	*P*-value
*Measured 24 to 72 h postmortem*						
pH	5.59	5.59	0.00	(−0.02, 0.02)	0.01	0.90
Drip loss, %	3.63	4.07	−0.44	(−0.81, −0.06)	0.19	0.02
L*	47.88	47.57	0.31	(−0.09, 0.71)	0.20	0.13
a*	7.08	6.90	0.18	(−0.02, 0.37)	0.10	0.09
b*	3.26	3.07	0.19	(0.02, 0.36)	0.09	0.03
IMF^1^, %	2.41	1.98	0.43	(0.26, 0.59)	0.08	<0.01
Color (NPPC score)^2^	2.93	2.91	0.02	(−0.06, 0.11)	0.04	0.52
Marbling (NPPC score)^2^	2.22	1.95	0.27	(0.11, 0.43)	0.08	<0.01
Firmness (NPPC score)^2^	1.99	1.94	0.05	(0.00, 0.10)	0.03	0.06
WBSF^3^, N	44.6	47.2	−2.6	(−4.3, −0.8)	0.9	<0.01
Cooking loss, %	22.95	23.44	−0.49	(−1.12, 0.13)	0.32	0.12
*Measured 14 d postmortem*						
L*	51.65	51.31	0.34	(−0.09, 0.78)	0.22	0.12
a*	7.16	7.15	0.01	(−0.20, 0.23)	0.11	0.88
b*	4.30	4.06	0.24	(0.07, 0.42)	0.09	0.01
WBSF^3^, N	36.9	38.6	−1.7	(−3.2, −0.4)	0.7	0.01
Cooking loss, %	21.62	22.23	−0.61	(−1.25, 0.04)	0.33	0.07

^1^IMF, intramuscular fat determined with ether extraction.

^2^NPPC, National Pork Producer’s Council pork quality standards.

^3^WBSF, Warner-Bratzler shear force.

Loin pass rate, a measurement that factors in industry-driven thresholds that meat primal cuts must meet (or exceed) to qualify for premium-based systems, was numerically greater by a magnitude of 10.33 percentage units for barrows compared with gilts (Table 8). This was largely driven by differences in subjective marbling scores; however, barrows had greater percentages of loins meeting or exceeding subjective color scores of 3.0 and subjective firmness scores of 2.0 when compared with gilts. To our knowledge, this is the first study that has quantified a parameter like primal pass rate between barrows and gilts, yet several other studies (Boler et al., 2014; Overholt et al., 2016; Redifer et al., 2020) have previously reported quality advantages for lean meat primals from barrows when compared with those from gilts.

**Table 8.** Effect of sex (conventionally-raised market barrows versus conventionally-raised market gilts) on loin primal pass rate (measured 24 to 72 h postmortem)

	Barrows	Gilts	Effect (barrows − gilts)
Color (NPPC^1^ score ≥ 3), % of population	90.48	84.57	5.91
Marbling (NPPC^1^ score ≥ 2), % of population	79.76	69.71	10.05
Firmness (NPPC^1^ score ≥ 2), % of population	98.21	91.43	6.78
Pass rate^2^, % of population	72.62	62.29	10.33

^1^NPPC = National Pork Producer’s Council pork quality standards.

^2^Pass rate is defined as loin primals that would have met quality thresholds set for color (≥3), marbling (≥2), and firmness (≥2).

### Interactions Between Sex and Hot Carcass Weight Quantiles

There were limited significant interactions between sex and hot carcass weight quantile in this study; therefore, the interaction means were presented in supplementary tables (Supplementary Tables S1 to S8) and the main effects were the focus of the above discussion.

There was a significant interaction between sex and hot carcass weight quantile for the 408B whole belly (skin-on, untrimmed, bone-in belly) when expressed as a percentage of chilled side weight (*P* = 0.05), which was caused by bellies increasing in their percentage of chilled side weight when barrows became heavier, and bellies expressed as a percentage of chilled side weight remaining unchanged when gilts became heavier. There were significant interactions between sex and hot carcass weight quantile for the weight (*P* = 0.02) and percentage of chilled side weight (*P* = 0.01) for the 416 spareribs (untrimmed spareribs). In both instances, the interactions were difficult to fully explain, yet it should be mentioned that there were no significant effects for sex for either parameter. There was a significant interaction between sex and hot carcass weight quantile for marbling of pork loins (*P* = 0.01), which was caused by barrows in the average hot carcass weight quantile (104 to 110 kg) having greater levels of marbling than gilts in the average hot carcass weight quantile (104 to 110 kg), while marbling was not different (*P* > 0.05) between barrows and gilts in both the lightweight quantile (<104 kg) and heavyweight quantile (>110 kg). This appears to be an anomaly with this particular dataset and other published studies do not support the interaction observed here between sex and carcass weight but would support differences in marbling between barrows and gilts (Overholt et al., 2016; Price et al., 2019; Redifer et al., 2020).

## Conclusions

This study confirms that carcasses from gilts are leaner than carcasses from barrows and provide improved carcass cutting yields (1.52% when expressed as a merchandized-cut yield and 2.92% when expressed as a dissected carcass lean yield). On the other hand, loins from barrows are higher quality than loins from gilts (a difference of 10.33 percentage units for primal pass rate). This information is useful for multiple sectors of the pork industry and should be continuously monitored over time as genetics, nutrition, management strategies, and carcass weights change to meet industry goals.

## Supplementary Material

txad079_suppl_Supplementary_Tables
