# Supplementary material for: A comparison of carcass characteristics, carcass cutting yields, and meat quality of barrows and gilts
Source: Transl Anim Sci. 2023 Jul 13;7(1):txad079. doi: 10.1093/tas/txad079 (PMC10464715; doi:10.1093/tas/txad079)
Supplement: txad079_suppl_Supplementary_Tables [file txad079_suppl_supplementary_tables.docx]

**SUPPLEMENTARY TABLES**

| **Supplementary Table 1.** Effect of sex (conventionally-raised market barrows versus conventionally-raised market gilts) and hot carcass weight (HCW) on carcass characteristics. | | | | | | | | | | | | |
| --- | --- | --- | --- | --- | --- | --- | --- | --- | --- | --- | --- | --- |
|  | Barrows | | | Gilts | | |  |  |  | *P*-values | | |
|  | Light  (< 104 kg) | Average  (104 – 110 kg) | Heavy  (> 110 kg) | Light  (< 104 kg) | Average  (104 – 110 kg) | Heavy  (> 110 kg) |  | SEM |  | Sex | HCW | Sex × HCW |
| No. of observations | 52 | 56 | 60 | 63 | 53 | 59 |  |  |  |  |  |  |
| Hot carcass weight^1^, kg | 100.08^c^ | 107.41^b^ | 114.99^a^ | 99.69^c^ | 107.21^b^ | 114.32^a^ |  | 0.53 |  | 0.24 | < 0.01 | 0.86 |
| Chilled side weight, kg | 43.86^c^ | 47.29^b^ | 50.89^a^ | 44.00^c^ | 47.25^b^ | 50.70^a^ |  | 0.27 |  | 0.84 | < 0.01 | 0.70 |
| Backfat thickness (Destron probe^2^), mm | 17.62^abc^ | 18.63^ab^ | 19.30^a^ | 14.66^d^ | 15.83^cd^ | 16.52^bcd^ |  | 0.62 |  | < 0.01 | 0.01 | 0.98 |
| Muscle depth (Destron probe^2^), mm | 65.79^b^ | 68.61^ab^ | 69.44^a^ | 67.72^ab^ | 70.96^a^ | 70.12^a^ |  | 0.93 |  | 0.02 | < 0.01 | 0.59 |
| Predicted lean yield (Destron 1994 equation^2^), % | 61.41^bcd^ | 61.07^cd^ | 60.79^cd^ | 62.85^a^ | 62.44^ab^ | 62.06^abc^ |  | 0.28 |  | < 0.01 | 0.03 | 0.93 |
| Predicted lean yield (Destron 2023 equation^2^), % | 56.87^bc^ | 56.26^c^ | 55.88^c^ | 59.01^a^ | 58.20^ab^ | 57.80^ab^ |  | 0.42 |  | < 0.01 | 0.02 | 0.94 |
| Backfat thickness (ultrasound^3^), mm | 17.22^ab^ | 17.99^a^ | 18.65^a^ | 14.57^c^ | 15.22^c^ | 15.84^bc^ |  | 0.55 |  | < 0.01 | 0.03 | 0.98 |
| Muscle depth (ultrasound^3^), mm | 65.25^c^ | 66.88^c^ | 67.84^bc^ | 68.23^abc^ | 70.99^ab^ | 71.20^a^ |  | 1.02 |  | < 0.01 | 0.01 | 0.77 |
| Backfat thickness (ruler^4^), mm | 17.46^ab^ | 18.77^a^ | 19.61^a^ | 14.57^c^ | 15.38^bc^ | 16.46^bc^ |  | 0.72 |  | < 0.01 | 0.01 | 0.90 |
| Muscle depth (ruler^4^), mm | 67.28^c^ | 69.20^bc^ | 71.38^ab^ | 69.42^bc^ | 72.71^a^ | 73.65^a^ |  | 0.73 |  | < 0.01 | < 0.01 | 0.53 |
| Loin eye area (tracing^4^), cm^2^ | 52.66^d^ | 54.45^cd^ | 57.55^ab^ | 56.33^bc^ | 58.38^ab^ | 60.29^a^ |  | 0.80 |  | < 0.01 | < 0.01 | 0.66 |
| ^1^ Hot carcass weight was measured as a head-on weight. | | | | | | | | | | | | |
| ^2^ The optical probe used in this study was the Destron PG-100 (International Destron Technologies); predicted lean yield equations were based on the following references (CPC, 1994; Bohrer et al., 2023). | | | | | | | | | | | | |
| ^3^ The ultrasound technology used in this study was the AutoFom III (Frontmatec A/S). | | | | | | | | | | | | |
| ^4^ Ruler and tracing measurements were collected on chilled carcasses during fabrication. | | | | | | | | | | | | |

| **Supplementary Table 2.** Effect of sex (conventionally-raised market barrows versus conventionally-raised market gilts) and hot carcass weight (HCW) on ham primal cut-out values. | | | | | | | | | | | | |
| --- | --- | --- | --- | --- | --- | --- | --- | --- | --- | --- | --- | --- |
|  | Barrows | | | Gilts | | |  |  |  | *P*-values | | |
|  | Light  (< 104 kg) | Average  (104 – 110 kg) | Heavy  (> 110 kg) | Light  (< 104 kg) | Average  (104 – 110 kg) | Heavy  (> 110 kg) |  | SEM |  | Sex | HCW | Sex × HCW |
| Whole ham (401A), kg | 12.05^c^ | 12.76^b^ | 13.73^a^ | 12.31^c^ | 13.05^b^ | 13.97^a^ |  | 0.12 |  | < 0.01 | < 0.01 | 0.97 |
| % chilled side wt | 27.50^abc^ | 26.99^bc^ | 26.97^c^ | 27.99^a^ | 27.62^ab^ | 27.55^abc^ |  | 0.20 |  | < 0.01 | < 0.01 | 0.89 |
| Trimmed ham (401C), kg | 9.04^d^ | 9.52^c^ | 10.32^b^ | 9.49^c^ | 10.05^b^ | 10.71^a^ |  | 0.12 |  | < 0.01 | < 0.01 | 0.79 |
| % chilled side wt | 20.62^bc^ | 20.14^c^ | 20.28^c^ | 21.59^a^ | 21.25^ab^ | 21.13^ab^ |  | 0.23 |  | < 0.01 | 0.07 | 0.76 |
| Lean ham (402G + additional lean), kg | 7.67^d^ | 8.08^c^ | 8.76^b^ | 8.16^c^ | 8.65^b^ | 9.16^a^ |  | 0.12 |  | < 0.01 | < 0.01 | 0.64 |
| % chilled side wt | 27.50^abc^ | 26.99^bc^ | 26.97^c^ | 27.99^a^ | 27.62^ab^ | 27.55^abc^ |  | 0.20 |  | < 0.01 | < 0.01 | 0.89 |
| Ham dissected lean yield^1^, % | 63.50^b^ | 63.16^b^ | 63.83^b^ | 66.19^a^ | 66.21^a^ | 65.62^a^ |  | 0.55 |  | < 0.01 | 0.92 | 0.18 |
| ^1^ Ham dissected lean yield = [(lean from 402G ham + additional lean) / 401A ham] × 100. | | | | | | | | | | | | |

| **Supplementary Table 3.** Effect of sex (conventionally-raised market barrows versus conventionally-raised market gilts) and hot carcass weight (HCW) on shoulder primal cut-out values. | | | | | | | | | | | | | |
| --- | --- | --- | --- | --- | --- | --- | --- | --- | --- | --- | --- | --- | --- |
|  | Barrows | | | Gilts | | |  |  |  | *P*-values | | | |
|  | Light  (< 104 kg) | Average  (104 – 110 kg) | Heavy  (> 110 kg) | Light  (< 104 kg) | Average  (104 – 110 kg) | Heavy  (> 110 kg) |  | SEM |  | Sex | HCW | Sex × HCW |  |
| Whole shoulder (403 + neckbones), kg | 8.70^c^ | 9.43^b^ | 10.16^a^ | 8.57^c^ | 9.38^b^ | 10.00^a^ |  | 0.08 |  | 0.03 | < 0.01 | 0.66 |  |
| % chilled side wt | 19.85^ab^ | 19.94^a^ | 19.94^a^ | 19.50^b^ | 19.86^ab^ | 19.70^ab^ |  | 0.12 |  | 0.01 | 0.12 | 0.43 |  |
| Picnic (405), kg | 4.45^c^ | 4.78^b^ | 5.14^a^ | 4.41^c^ | 4.77^b^ | 5.09^a^ |  | 0.05 |  | 0.32 | < 0.01 | 0.89 |  |
| % chilled side wt | 10.14 | 10.11 | 10.09 | 10.03 | 10.10 | 10.04 |  | 0.08 |  | 0.28 | 0.88 | 0.75 |  |
| Boneless picnic (405A), kg | 4.01^c^ | 4.30^b^ | 4.64^a^ | 4.04^c^ | 4.37^b^ | 4.65^a^ |  | 0.05 |  | 0.19 | < 0.01 | 0.74 |  |
| % chilled side wt | 9.15 | 9.10 | 9.11 | 9.19 | 9.25 | 9.17 |  | 0.09 |  | 0.12 | 0.89 | 0.69 |  |
| Butt (406), kg | 4.27^c^ | 4.65^b^ | 5.02^a^ | 4.17^c^ | 4.61^b^ | 4.90^a^ |  | 0.05 |  | < 0.01 | < 0.01 | 0.59 |  |
| % chilled side wt | 9.74^ab^ | 9.83^a^ | 9.87^a^ | 9.48^b^ | 9.75^ab^ | 9.68^ab^ |  | 0.09 |  | < 0.01 | 0.03 | 0.44 |  |
| Boneless butt (406A), kg | 3.39^c^ | 3.66^b^ | 3.96^a^ | 3.41^c^ | 3.73^b^ | 3.98^a^ |  | 0.04 |  | 0.16 | < 0.01 | 0.56 |  |
| % chilled side wt | 7.72 | 7.73 | 7.79 | 7.75 | 7.90 | 7.85 |  | 0.08 |  | 0.09 | 0.39 | 0.47 |  |
| Picnic dissected lean yield^1^, % | 67.08^b^ | 66.53^b^ | 67.00^b^ | 69.86^a^ | 69.73^a^ | 69.22^a^ |  | 0.49 |  | < 0.01 | 0.65 | 0.43 |  |
| Butt dissected lean yield^2^, % | 63.64^b^ | 62.46^b^ | 63.13^b^ | 67.55^a^ | 66.56^a^ | 66.44^a^ |  | 0.76 |  | < 0.01 | 0.19 | 0.75 |  |
| Shoulder dissected lean yield^3^, % | 61.78^b^ | 61.00^b^ | 61.65^b^ | 64.74^a^ | 64.47^a^ | 64.04^a^ |  | 0.53 |  | < 0.01 | 0.47 | 0.40 |  |
| ^1^ Picnic dissected lean yield = (lean from 405A boneless picnic / 405 picnic) × 100. | | | | | | | | | | | | | |
| ^2^ Butt dissected lean yield = (lean from 406A boneless butt / 406 butt) × 100. | | | | | | | | | | | | | |
| ^3^ Shoulder dissected lean yield = [(lean from 405A boneless picnic + lean from 406A boneless butt + lean from neckbones) / (403 shoulder + neckbones)] × 100. | | | | | | | | | | | | | |

| **Supplementary Table 4.** Effect of sex (conventionally-raised market barrows versus conventionally-raised market gilts) and hot carcass weight (HCW) on belly primal cut-out values. | | | | | | | | | | | | | |
| --- | --- | --- | --- | --- | --- | --- | --- | --- | --- | --- | --- | --- | --- |
|  | Barrows | | | Gilts | | |  |  |  | *P*-values | | |  |
|  | Light  (< 104 kg) | Average  (104 – 110 kg) | Heavy  (> 110 kg) | Light  (< 104 kg) | Average  (104 – 110 kg) | Heavy  (> 110 kg) |  | SEM |  | Sex | HCW | Sex × HCW |  |
| Whole belly (408B), kg | 7.92^c^ | 8.78^b^ | 9.43^a^ | 8.07^c^ | 8.62^b^ | 9.25^a^ |  | 0.11 |  | 0.31 | < 0.01 | 0.06 |  |
| % chilled side wt | 18.02 | 18.56 | 18.54 | 18.30 | 18.24 | 18.25 |  | 0.19 |  | 0.34 | 0.22 | 0.05 |  |
| Boneless belly (408), kg | 5.80^c^ | 6.46^b^ | 7.05^a^ | 5.85^c^ | 6.34^b^ | 6.82^a^ |  | 0.09 |  | 0.08 | < 0.01 | 0.16 |  |
| % chilled side wt | 13.21^b^ | 13.67^ab^ | 13.83^a^ | 13.28^b^ | 13.41^ab^ | 13.45^ab^ |  | 0.17 |  | 0.08 | 0.02 | 0.21 |  |
| Spareribs (416), kg | 1.33^c^ | 1.46^ab^ | 1.53^a^ | 1.36^c^ | 1.40^bc^ | 1.52^a^ |  | 0.03 |  | 0.31 | < 0.01 | 0.02 |  |
| % chilled side wt | 3.01 | 3.09 | 3.01 | 3.08 | 2.96 | 3.00 |  | 0.05 |  | 0.35 | 0.54 | 0.01 |  |
| Belly dissected lean yield^1^, % | 52.55^ab^ | 50.26^b^ | 50.70^b^ | 54.45^a^ | 53.79^a^ | 52.04^ab^ |  | 0.84 |  | < 0.01 | 0.01 | 0.20 |  |
| ^1^ Belly dissected lean yield = [(lean from 409A belly + lean from 416 spareribs) / 408B belly] × 100. | | | | | | | | | | | | | |

| **Supplementary Table 5.** Effect of sex (conventionally-raised market barrows versus conventionally-raised market gilts) and hot carcass weight (HCW) on loin primal cut-out values. | | | | | | | | | | | | | |
| --- | --- | --- | --- | --- | --- | --- | --- | --- | --- | --- | --- | --- | --- |
|  | Barrows | | | Gilts | | |  |  |  | *P*-values | | |  |
|  | Light  (< 104 kg) | Average  (104 – 110 kg) | Heavy  (> 110 kg) | Light  (< 104 kg) | Average  (104 – 110 kg) | Heavy  (> 110 kg) |  | SEM |  | Sex | HCW | Sex × HCW |  |
| Whole loin, skin-on (410), kg | 12.72^c^ | 13.75^b^ | 14.92^a^ | 12.60^c^ | 13.64^b^ | 14.78^a^ |  | 0.11 |  | 0.13 | < 0.01 | 0.99 |  |
| % chilled side wt | 29.08 | 29.06 | 29.23 | 28.69 | 28.87 | 29.04 |  | 0.20 |  | 0.07 | 0.33 | 0.75 |  |
| Whole loin, skinless (410), kg | 9.93^d^ | 10.64^c^ | 11.52^a^ | 10.23^d^ | 10.99^b^ | 11.82^a^ |  | 0.09 |  | < 0.01 | < 0.01 | 0.93 |  |
| % chilled side wt | 22.69^bc^ | 22.49^c^ | 22.60^c^ | 23.30^a^ | 23.28^ab^ | 23.28^ab^ |  | 0.16 |  | < 0.01 | 0.73 | 0.83 |  |
| Boneless sirloin (413D), kg | 1.35^de^ | 1.50^bc^ | 1.64^a^ | 1.32^e^ | 1.47^cd^ | 1.60^ab^ |  | 0.03 |  | 0.12 | < 0.01 | 0.98 |  |
| % chilled side wt | 3.09 | 3.17 | 3.21 | 3.01 | 3.10 | 3.15 |  | 0.07 |  | 0.13 | 0.09 | 0.99 |  |
| Canadian back loin (414), kg | 3.39^d^ | 3.58^c^ | 3.85^b^ | 3.59^c^ | 3.87^b^ | 4.08^a^ |  | 0.05 |  | < 0.01 | < 0.01 | 0.64 |  |
| % chilled side wt | 7.75^bc^ | 7.57^c^ | 7.55^c^ | 8.17^a^ | 8.19^a^ | 8.03^ab^ |  | 0.10 |  | < 0.01 | 0.21 | 0.57 |  |
| Tenderloin (415), kg | 0.81^d^ | 0.87^c^ | 0.96^a^ | 0.81^d^ | 0.90^bc^ | 0.95^ab^ |  | 0.02 |  | 0.59 | < 0.01 | 0.40 |  |
| % chilled side wt | 1.86 | 1.85 | 1.88 | 1.85 | 1.90 | 1.87 |  | 0.03 |  | 0.50 | 0.60 | 0.36 |  |
| Back ribs (422), kg | 2.21^c^ | 2.32^b^ | 2.45^a^ | 2.23^bc^ | 2.31^b^ | 2.52^a^ |  | 0.03 |  | 0.11 | < 0.01 | 0.26 |  |
| % chilled side wt | 5.04^a^ | 4.90^ab^ | 4.82^b^ | 5.08^a^ | 4.89^ab^ | 4.97^ab^ |  | 0.06 |  | 0.11 | < 0.01 | 0.22 |  |
| Loin dissected lean yield^1^, % | 53.78^b^ | 52.95^b^ | 52.90^b^ | 57.48^a^ | 57.44^a^ | 56.43^a^ |  | 0.07 |  | < 0.01 | 0.32 | 0.66 |  |
| ^1^ Loin dissected lean yield = [(lean from 413D sirloin + lean from 414 Canadian back loin + lean from 415 tenderloin + lean from 422 back ribs) / 410 loin skin-on] × 100. | | | | | | | | | | | | | |

| **Supplementary Table 6.** Effect of sex (conventionally-raised market barrows versus conventionally-raised market gilts) and hot carcass weight (HCW) on carcass cutting yields. | | | | | | | | | | | | | |
| --- | --- | --- | --- | --- | --- | --- | --- | --- | --- | --- | --- | --- | --- |
|  | Barrows | | | Gilts | | |  |  |  | *P*-values | | |  |
|  | Light  (< 104 kg) | Average  (104 – 110 kg) | Heavy  (> 110 kg) | Light  (< 104 kg) | Average  (104 – 110 kg) | Heavy  (> 110 kg) |  | SEM |  | Sex | HCW | Sex × HCW |  |
| Primal-cut yield^1^, % | 94.45^b^ | 94.55^ab^ | 94.73^a^ | 94.46^b^ | 94.57^ab^ | 94.63^ab^ |  | 0.07 |  | 0.58 | < 0.01 | 0.37 |  |
| Merchandized-cut yield^2^, % | 68.29^b^ | 68.18^b^ | 68.44^b^ | 69.94^a^ | 69.93^a^ | 69.59^a^ |  | 0.29 |  | < 0.01 | 0.92 | 0.34 |  |
| Dissected carcass lean yield^3^, % | 55.92^bc^ | 54.98^c^ | 55.42^c^ | 58.74^a^ | 58.59^a^ | 57.74^ab^ |  | 0.58 |  | < 0.01 | 0.32 | 0.33 |  |
| ^1^ Primal-cut yield = [(401A ham + 405 picnic + 406 butt + 408B belly + 410 loin) / chilled side weight] × 100. | | | | | | | | | | | | | |
| ^2^ Merchandized-cut yield = {[(402G ham + additional lean) + 405A picnic + 406A butt + 408 belly + 416 spareribs + 413D sirloin + 414 Canadian back loin + 415 tenderloin + 422 back ribs] / chilled side weight} × 100. | | | | | | | | | | | | | |
| ^3^ Dissected carcass lean yield = [(dissected lean from ham + dissected lean from picnic + dissected lean from butt + dissected lean from belly + dissected lean from loin + dissected lean from ribs and neckbones) / chilled side weight] × 100. | | | | | | | | | | | | | |

| **Supplementary Table 7.** Effect of sex (conventionally-raised market barrows versus conventionally-raised market gilts) and hot carcass weight (HCW) on meat quality traits. | | | | | | | | | | | | |
| --- | --- | --- | --- | --- | --- | --- | --- | --- | --- | --- | --- | --- |
|  | Barrows | | | Gilts | | |  |  |  | *P*-values | | |
|  | Light  (< 104 kg) | Average  (104 – 110 kg) | Heavy  (> 110 kg) | Light  (< 104 kg) | Average  (104 – 110 kg) | Heavy  (> 110 kg) |  | SEM |  | Sex | HCW | Sex × HCW |
| ***Measured 24-72 hours post-mortem*** | | | | | | | | | | | | |
| pH | 5.59 | 5.59 | 5.59 | 5.59 | 5.60 | 5.59 |  | 0.02 |  | 0.90 | 0.79 | 0.84 |
| Drip loss, % | 3.49 | 3.43 | 3.97 | 3.96 | 4.13 | 4.12 |  | 0.35 |  | 0.02 | 0.48 | 0.50 |
| *L** | 48.08 | 47.92 | 47.65 | 47.80 | 47.50 | 47.40 |  | 0.3 |  | 0.13 | 0.36 | 0.94 |
| *a** | 7.13^a^ | 7.03^ab^ | 7.06^a^ | 6.63^b^ | 6.90^ab^ | 7.19^a^ |  | 0.2 |  | 0.09 | 0.27 | 0.04 |
| *b** | 3.30 | 3.20 | 3.30 | 2.94 | 3.09 | 3.20 |  | 0.2 |  | 0.03 | 0.55 | 0.40 |
| IMF^1^, % | 2.34^ab^ | 2.50^a^ | 2.40^ab^ | 1.98^b^ | 1.96^b^ | 2.01^b^ |  | 0.18 |  | < 0.01 | 0.82 | 0.64 |
| Color (NPPC score)^2^ | 2.95 | 2.98 | 2.88 | 2.91 | 2.86 | 2.95 |  | 0.06 |  | 0.52 | 0.96 | 0.19 |
| Marbling (NPPC score)^2^ | 2.00^b^ | 2.46^a^ | 2.20^ab^ | 1.84^b^ | 1.82^b^ | 2.19^ab^ |  | 0.14 |  | < 0.01 | 0.05 | 0.01 |
| Firmness (NPPC score)^2^ | 1.98 | 2.00 | 2.00 | 1.95 | 1.91 | 1.96 |  | 0.04 |  | 0.06 | 0.79 | 0.64 |
| WBSF^3^, N | 44.6^b^ | 45.1^ab^ | 44.2^b^ | 49.2^a^ | 46.1^ab^ | 46.2^ab^ |  | 1.7 |  | < 0.01 | 0.39 | 0.24 |
| Cooking loss, % | 23.27 | 23.29 | 22.29 | 23.42 | 23.23 | 23.66 |  | 0.95 |  | 0.12 | 0.72 | 0.14 |
| ***Measured 14 days post-mortem*** | | | | | | | | | | | | |
| *L** | 51.79 | 51.79 | 51.38 | 51.49 | 51.36 | 51.08 |  | 0.4 |  | 0.12 | 0.39 | 0.96 |
| *a** | 7.25 | 7.11 | 7.13 | 7.04 | 7.15 | 7.26 |  | 0.2 |  | 0.88 | 0.89 | 0.42 |
| *b** | 4.33 | 4.28 | 4.30 | 4.00 | 4.08 | 4.09 |  | 0.2 |  | 0.01 | 0.98 | 0.81 |
| WBSF^3^, N | 36.6 | 36.5 | 37.6 | 38.9 | 38.3 | 38.6 |  | 1.2 |  | 0.01 | 0.77 | 0.73 |
| Cooking loss, % | 22.06 | 20.97 | 21.84 | 22.10 | 22.22 | 22.38 |  | 1.13 |  | 0.07 | 0.41 | 0.34 |
| ^1^ IMF = intramuscular fat determined with ether extraction. | | | | | | | | | | | | |
| ^2^ NPPC = National Pork Producer’s Council pork quality standards. | | | | | | | | | | | | |
| ^3^ WBSF = Warner-Bratzler shear force. | | | | | | | | | | | | |

| **Supplementary Table 8.** Effect of sex (conventionally-raised market barrows versus conventionally-raised market gilts) and hot carcass weight (HCW) on loin primal pass rate (measured 24 to 72 hours postmortem). | | | | | | |
| --- | --- | --- | --- | --- | --- | --- |
|  | Barrows | | | Gilts | | |
|  | Light  (< 104 kg) | Average  (104 – 110 kg) | Heavy  (> 110 kg) | Light  (< 104 kg) | Average  (104 – 110 kg) | Heavy  (> 110 kg) |
| Color (NPPC^1^ score ≥ 3), % of population | 92.31 | 94.64 | 85.00 | 88.89 | 77.36 | 86.44 |
| Marbling (NPPC^1^ score ≥ 2), % of population | 73.08 | 85.71 | 80.00 | 71.43 | 60.38 | 76.27 |
| Firmness (NPPC^1^ score ≥ 2), % of population | 98.08 | 98.21 | 98.33 | 92.06 | 86.79 | 94.92 |
| Pass rate^2^, % of population | 69.23 | 80.36 | 68.33 | 69.84 | 49.06 | 66.10 |
| ^1^ NPPC = National Pork Producer’s Council pork quality standards. | | | | | | |
| ^2^ Pass rate is defined as loin primals that would have met quality thresholds set for color (≥ 3), marbling (≥ 2), and firmness (≥ 2). | | | | | | |
